# Cas12a-mediated gene targeting by sequential transformation strategy in *Arabidopsis thaliana*

**DOI:** 10.1186/s12870-024-05375-z

**Published:** 2024-07-12

**Authors:** Jing Li, Qi Wei, Yiqiu Cheng, Dali Kong, Zhe Kong, Yongping Ke, Xiaofei Dang, Jian-Kang Zhu, Hiroaki Shimada, Daisuke Miki

**Affiliations:** 1 Shanghai Center for Plant Stress Biology, CAS Center for Excellence in Molecular Plant Sciences, Chinese Academy of Sciences, Shanghai, 200032 China; 2 University of Chinese Academy of Sciences, Beijing, 100049 China; 3 Institute of Advanced Biotechnology and School of Medicine, Southern University of Science and Technology, Shenzhen, 518055 China; 4 Department of Biological Science and Technology, Tokyo University of Science, Katsushika, Tokyo 125-8585 Japan

**Keywords:** Genome engineering, Gene targeting, Cas12a, Enhancer, Sequential transformation, *Arabidopsis thaliana*

## Abstract

Gene targeting (GT) allows precise manipulation of genome sequences, such as knock-ins and sequence substitutions, but GT in seed plants remains a challenging task. Engineered sequence-specific nucleases (SSNs) are known to facilitate GT via homology-directed repair (HDR) in organisms. Here, we demonstrate that Cas12a and a temperature-tolerant Cas12a variant (ttCas12a) can efficiently establish precise and heritable GT at two loci in *Arabidopsis thaliana* (Arabidopsis) through a sequential transformation strategy. As a result, ttCas12a showed higher GT efficiency than unmodified Cas12a. In addition, the efficiency of transcriptional and translational enhancers for GT via sequential transformation strategy was also investigated. These enhancers and their combinations were expected to show an increase in GT efficiency in the sequential transformation strategy, similar to previous reports of all-in-one strategies, but only a maximum twofold increase was observed. These results indicate that the frequency of double strand breaks (DSBs) at the target site is one of the most important factors determining the efficiency of genetic GT in plants. On the other hand, a higher frequency of DSBs does not always lead to higher efficiency of GT, suggesting that some additional factors are required for GT via HDR. Therefore, the increase in DSB can no longer be expected to improve GT efficiency, and a new strategy needs to be established in the future. This research opens up a wide range of applications for precise and heritable GT technology in plants.

**Supplementary Information:**

The online version contains supplementary material available at 10.1186/s12870-024-05375-z.

## Key message

The combination of enhancers, Cas12a, and sequential transformation strategies enables efficient and precise heritable gene targeting in Arabidopsis.

**Supplementary Information:**

The online version contains supplementary material available at 10.1186/s12870-024-05375-z.

## Background

Engineered sequence-specific nucleases (SSNs) can generate target site-specific double-strand breaks (DSBs) in the genomes of many organisms [1, 2]. Currently, the clustered regularly interspaced short palindromic repeats (CRISPR)/CRISPR-associated protein (Cas) system, one of the engineered SSNs, is the most widely used for genome editing in many organisms, including plants, because of its simplicity, high specificity, and high efficiency [3–5]. DSBs generated by these SSNs are repaired predominantly by the error-prone non-homologous end-joining (NHEJ) pathway, but rarely by error-free homology-directed repair (HDR) if an appropriate donor template is supplied [6, 7]. HDR-mediated gene targeting (GT) can create desirable sequence alterations such as precise sequence knock-ins (KIs) or substitutions in the genome. Therefore, GT is a powerful tool for molecular research and multiple biotechnological applications, and is being used in a wide variety of organisms [3, 8, 9]. However, even though SSNs are able to promote GT efficiency in organisms, GT remains a challenging task in seed plants due to the extremely low frequency of HDR and the difficulty of delivering donor templates [6, 10].

The most commonly used CRISPR/Cas9 system in plants is Type II *Streptococcus pyogenes* Cas9 (SpCas9: hereafter Cas9). Cas9 recognizes a specific G-rich (NGG) protospacer adjacent motif (PAM) sequence and cleaves the proximal end of 3–4 base pairs of PAM to generate the blunt end of the DSB. When the genomic site of interest is AT-rich, it is difficult to design appropriate sgRNAs for Cas9. Therefore, in order to establish GT at a wide range of target sites, this study examined another popular Type V CRISPR/Cas system, the *Lachnospiraceae bacterium ND2006* Cas12a (LbCas12a: hereafter Cas12a). Cas12a recognizes T-rich TTTV (V = A/G/C) PAM sequences and generates a 5' overhang staggered end DSB on the distal side of the PAM. It is thought that Cas12a generates the staggered end of the DSB distal to the PAM sequence, which may facilitate repetitive cleavage and extensive end processing, potentially increasing the efficiency of GT [11]. However, Cas12a had low enzyme activity at low temperatures, which is mandatory for plant cultivation. To overcome the reduced enzymatic activity of Cas12a in plants, a temperature tolerant LbCas12a variant (ttLbCas12a; hereafter ttCas12a) with a single D156R mutation was developed [12]. In addition, the all-in-one strategy via ttCas12a has successfully improved GT efficiency in Arabidopsis [13].

The employment of transcriptional or translational enhancers for SSNs is an alternative approach to improve the frequency of DSB in plants. Using the 5' UTR containing the first intron of Arabidopsis *Ubiquitin 10* (AtUbq10) as a transcriptional enhancer, Cas9-mediated heritable mutants were generated in barley with high efficiency [14]. Similarly, the intron-containing version of Cas9 showed a high mutation frequency in plants [15]. In addition, the use of dMac3, a highly efficient translational enhancer of the rice *OsMac3* gene, increased the efficiency of Cas9- and TALEN-mediated mutagenesis in plants including tetraploid potato [16–20]. Such transcriptional and translational enhancers have been used extensively for mutagenesis purposes, but their application to HDR-mediated GT has been rare. The application of the omega translational enhancer from tobacco mosaic virus (TMV) to Cas9 has been reported to improve the all-in-one strategy GT in Arabidopsis [21]. Furthermore, during preparation of this manuscript, an intron-containing version of ttCas12a was reported to promote DSB and GT efficiency in Arabidopsis [22].

Recently, significant progress and successful GT via HDR using engineered SSNs has been reported in several plant species, such as Arabidopsis [22–26], soybean [27], rice [28–31], maize [32], wheat [33], and poplar [34, 35]. However, most of these methods relied on the selection of antibiotic markers or herbicide resistance genes at target loci. We have reported a sequential transformation strategy for efficient CRISPR/Cas9-mediated GT in Arabidopsis and rice [31, 36, 37]. As a brief overview, constructs with donor and sgRNA are transformed into parental lines that stably express Cas9 in egg cells and early embryos by the DD45 promoter in Arabidopsis. Although the efficiency of GT with a sequential transformation strategy using Cas9 is higher than the all-in-one approach, the application of the Cas12a system to a sequential transformation strategy has not been explored to date. Furthermore, it has not been investigated whether the combination with enhancers can elevate GT efficiency in a sequential transformation strategy. The characteristics of Cas12a, the use of enhancers, and the sequential transformation strategy were expected to increase GT efficiency. Here, we demonstrated that in Arabidopsis, the combination of enhancers increases the efficiency of GT through a sequential transformation strategy mediated by both Cas12a and ttCas12a. The data indicated that ttCas12a exhibited greater GT efficiency than Cas12a. Furthermore, the application of enhancers was found to result in an improvement in GT efficiency, although not always. These results indicate that DSB frequency is one of the most important factors determining the efficiency of SSN-mediated GT. However, the GT efficiencies obtained in this study were not as high as we had anticipated. The results of this study have made it possible to establish efficient and precise GT in a wider range of target sequences.

## Results

### Preparation of Cas12a parental lines with and without enhancers for efficient GT establishment

**Fig. 1:**
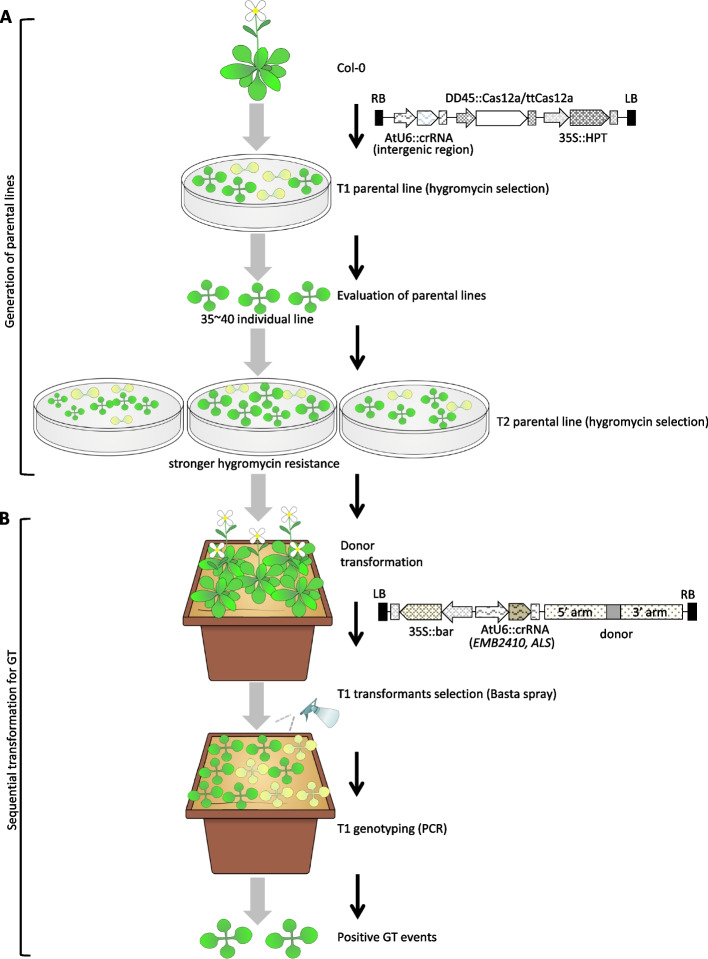
Overview of sequential transformation strategies for Cas12a/ttCas12a-mediated GT establishment. **A** Generation of parental lines. Parental line construct contains a Cas12a/ttCas12a cassette driven by the DD45 promoter, a crRNA targeting the intergenic region driven by the AtU6 promoter, and a hygromycin selection marker gene cassette driven by the 35S promoter. Cas12a/ttCas12a expression constructs were transformed into Col-0 accession by Agrobacterium to generate parental lines. Screening of T1 transgenic parental lines with 50 mg/L hygromycin yielded approximately 35–40 individual lines. To evaluate the obtained parental line candidates, mutation frequencies in the target intergenic region, Cas12a/ttCas12a copy number, and hygromycin-resistant phenotype of the progenies were examined. **B** Sequential transformation strategy for GT establishment. Parental lines with greater hygromycin resistance, higher mutation frequency, and lower copy number of Cas12a/ttCas12a were used for sequential transformations. The donor constructs used for sequential transformation contain a crRNA cassette at the target locus driven by the AtU6 promoter, a repair donor template for the HDR, and a cassette of herbicide resistance marker gene *bar* driven by the 35S promoter. T1 transformants of the donor constructs were first screened with Basta spray and then genotyped to obtain GT-positive events

To evaluate the double-strand break (DSB) activity of the CRISPR/Cas12a systems, mutation frequencies were examined. Because the Cas12a transgenic plants generated will be used as parental lines for gene targeting (GT) via sequential transformation (Fig. 1), a CRISPR RNA (crRNA) was designed to be located between At1g53990 and At1g54000, where these two genes are positioned in the tail-to-tail direction (Supplementary Figure S1). Theoretically, it is highly unlikely that mutations in this intergenic region would affect expression of genes or stability of genome [31]. Previous reports have used the Cas9 parental line harboring an sgRNA targeting the *GLABRA2* (*GL2*) gene for efficient GT in *Arabidopsis thaliana* (Arabidopsis) [25, 36, 37]. While *gl2* mutations are useful as a visible marker [38], trichome formation often affects plant physiological functions, such as biotic and abiotic stress responses [39, 40]. Although mutations at the *GL2* locus in Cas9 parental lines can be removed by backcrossing [37], undesirable *gl2* mutations frequently prevent rapid study execution. Therefore, in this study, the intergenic region was chosen as the target of crRNA in the parental lines (Fig. 1A, Supplementary Figure S1).

**Fig. 2:**
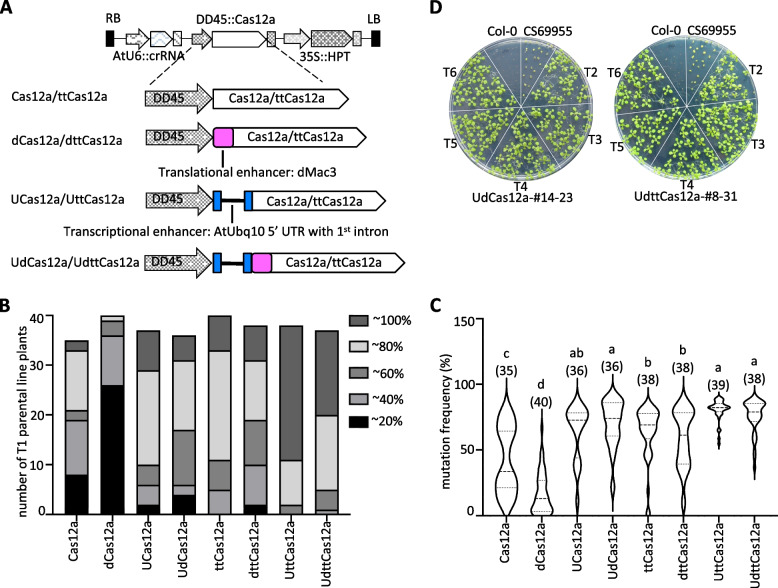
Evaluation of Cas12a/ttCas12a parental lines in Arabidopsis. **A** Schematic diagram of parental line constructs. Cas12a/ttCas12 represents a parental construct containing without enhancer, dCas12a/dttCas12 represents a parental construct containing the dMac3 translational enhancer, UCas12a/UttCas12a represents a parental construct containing the AtUbq10 transcriptional enhancer, and UdCas12a/UdttCas12 represents a parental construct containing both enhancers. The blue rectangular block and black line represent the 5' UTR sequence transcriptional enhancer of AtUbq10 with the first intron. Magenta square indicates the dMac3 translational enhancer. **B** Proportion of mutation frequencies in individual T1 generations of the eight parental line constructs. Mutation frequency of intergenic target site was determined by T7EI digestion assay. **C** Statistical analysis of mutation frequency in parental lines. The numbers in parenthesis represent the number of individual samples analyzed. One-way ANOVA and Tukey test were applied to analyze standard differences (*P* < 0.05). **D** Hygromycin-resistant phenotypes in different generations. Homozygous transgenic lines of UdCas12a-#14–23 and UdttCas12a-#8–31 were selected in the T2 to T6 generation (Supplementary Figure S4). Seeds were germinated on 1/2 MS plates containing 50 mg/L hygromycin. Col-0 and CS69955 (previously reported Cas9 parental line) were used as controls

To gain a comprehensive understanding, we conducted an investigation into the most effective means of establishing GT in Arabidopsis. This involved the use of transcriptional and translational enhancers, both alone and in combination. In addition to Cas12a, temperature tolerant Cas12a (ttCas12a), which exhibits higher DSB activity and GT efficiency [12, 13], was investigated in the present study (Fig. 2A). A total eight constructs for the parental lines were generated and examined. The constructs without enhancers were used as controls (Cas12a and ttCas12a). The constructs with enhancers were: translational enhancer dMac3 alone (dCas12a and dttCas12a), transcriptional enhancer Arabidopsis *Ubiquitin 10* (AtUbq10) first intron alone (UCas12a and UttCas12a), and the combination of them (UdCas12a and UdttCas12a). Based on previous reports, we applied the egg cell and early embryo specific DD45 promoter to all constructs [21, 25, 36, 37] (Fig. 2A).

The mutation frequencies were measured in 35 to 40 independent T1 transgenic plants for all eight constructs. The results showed that ttCas12a exhibited higher mutational frequency than Cas12a in all cases except the combination of the two enhancers (UdCas12a) (Fig. 2B, C, Supplementary Figure S2, S3), consistent with previous reports [12]. The combination of AtUbq10 and dMac3 significantly increased mutation frequency in both Cas12a and ttCas12a compared to controls or the use of a single dMac3 enhancer alone (Fig. 2C). These mutational frequencies at the target locus appear to be nearly saturated when the transcriptional enhancer AtUbq10 is used alone or in combination with the two enhancers. In contrast, the translational enhancer dMac3 did not necessarily increase Cas12a mutation frequency, which was lower than in the control without the enhancer (Fig. 2C). Furthermore, the results suggested that there is no statistical correlation between the number of copies of Cas12a or ttCas12a and mutation frequency in T1 plants (Supplementary Figure S2, S3) [31].

In the sequential transformation strategy, the DSB activity of the Cas protein of the parental lines must be one of the key factors that determine GT efficiency. Silencing of DD45 pro::Cas9 transgene activity in the parental line (ABRC stock CS69955) has been reported, resulting in reduced GT efficiency [37]. To avoid transgene silencing, Cas12a and ttCas12a candidate parental lines were selected with hygromycin at a concentration of 50 mg/L until T6 generation (Fig. 2D, Supplementary Figure S4). Three independent lines for each construct were selected as parental lines for subsequent studies according to mutation rate and hygromycin resistance (Supplementary Figure S2, S3, S4). The efficiency of GT was anticipated to be enhanced by applying the enhancers alone or in combination.

### *GFP* knock-in at the *EMB2410* locus

**Fig. 3:**
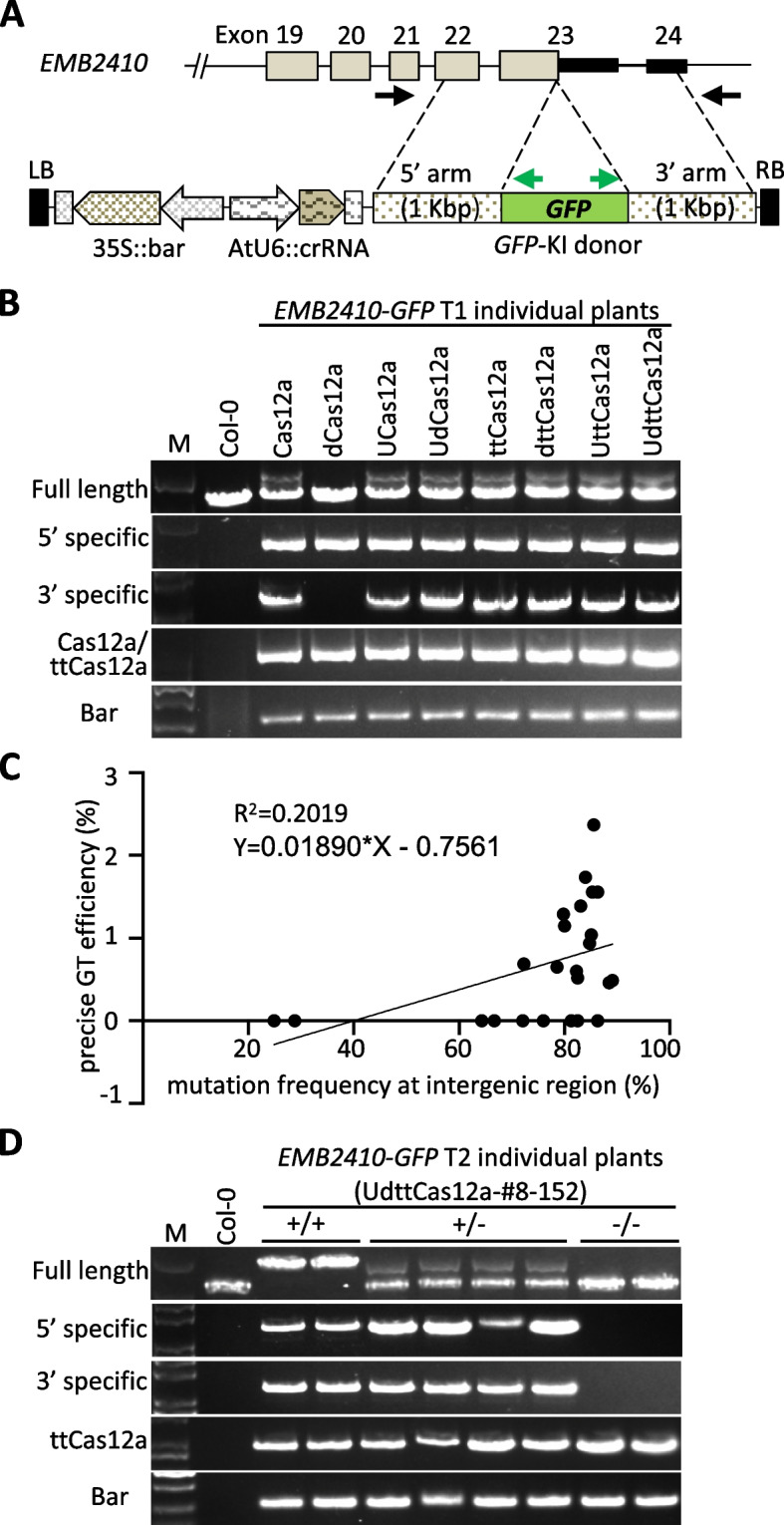
Characterization of *GFP*-KI events at the *EMB2410* locus. **A** Diagram of donor construct for sequential transformation. The donor template has a *GFP*-KI fragment with 1 Kbp long homology arms at the *EMB2410* target locus. Black arrows indicate full-length primers used for genotyping, green arrows in *GFP* fragments indicate specific primers. **B** PCR genotyping of precise *GFP*-KI at the *EMB2410* target locus in T1 transformants. **C** Correlation analysis of mutation frequency at the target intergenic region in parental lines and precise *GFP*-KI GT efficiency at the *EMB2410* locus. **D** Heritable *GFP*-KI GT in UdttCas12a-#8–152 T2 generation. Col-0 was used as a control. M; DNA size marker

Precise *GFP* knock-in (KI) at the *Embryo Defective 2410* (*EMB2410*) locus was successfully achieved by a Cas9-mediated sequential transformation strategy [37, 25]. In the present study, the same *GFP*-KI donor sequence with 1 Kbp homology arms was applied. A sequential transformation construct with an AtU6-26 promoter-driven crRNA and *GFP*-KI donor targeting the *EMB2410* locus was constructed and transformed into a total of 24 Cas12a and ttCas12a parental lines (Figs. 1B, 3A). T1 transgenic plants were screened by Basta spraying and then GT events were determined by PCR for all Basta-resistant T1 plants (Fig. 1B). To analyze GT events, three different primer sets were designed for PCR based genotyping (Fig. 3A). The 5' and 3' arms specific primers were designed to specifically detect *GFP*-KI events. The last one, full-length primers were designed to anneal to the upstream and downstream of the homology arms, capable of amplifying both endogenous and precise GT alleles. The specific primer sets were used for initial screening, and then all possible T1 candidate plants were characterized by using full-length primer set to assess precise GT. Previous reports indicate that all GT events obtained using full-length primer sets are accurate and stably inherited by progenies [25, 37, 31]. GT efficiency was calculated by the number of T1 transgenic plants examined (Table 1). As a result, at least one precise and heritable GT event was obtained in all parental line constructs except dCas12a (Fig. 3B, Table 1). All *EMB2410*-*GFP* KI events were confirmed by Sanger sequencing. The results showed that all *GFP*-KI GT events at the *EMB2410* locus detected by the full-length primer set were precise and seamless (Supplementary Figure S5A). In all combinations with and without enhancers, ttCas12a showed higher GT efficiency than Cas12a. The efficiency of the precise GT events was associated in the mutation frequency of the intergenic region of the parental lines (Fig. 3C). All precise *EMB2410*-*GFP* KI events detected with the full-length primer set were stably inherited by the progeny, in accordance with Mendelian ratio (Fig. 3D). These results collectively suggest that the CRISPR/Cas12a system with the AtUbq10 transcriptional enhancer represents a promising approach for the efficient generation of precise GT in plants [13, 41, 42].

**Table 1:** Knock-in GT efficiencies at the *EMB2410* locus mediated by Cas12a/ttCas12a

Parental line		Number of transformants analyzed	5' arm GT (Total HDR)	5' arm GT (Total HDR) efficiency (%)	Precise GT	Precise GT efficiency (%)	SDs (*P* < 0.05)
Cas12a	#5	281	4	1.42	0	0	b
	#30	194	2	1.03	0	0	
	#35	288	10	3.47	2	0.69	
dCas12a	#4	150	0	0	0	0	b
	#11	140	2	1.43	0	0	
	#12	148	1	0.68	0	0	
UCas12a	#24	191	5	2.62	1	0.52	ab
	#26	212	17	8.02	2	0.94	
	#27	199	9	4.52	0	0	
UdCas12a	#2	204	11	5.39	1	0.49	b
	#3	219	9	4.11	1	0.46	
	#14	162	2	1.23	0	0	
ttCas12a	#1	174	7	4.02	2	1.15	a
	#5	288	21	7.29	5	1.74	
	#9	233	17	7.30	3	1.29	
dttCas12a	#6	153	2	1.31	1	0.65	b
	#22	179	4	2.23	0	0	
	#28	163	4	2.45	0	0	
UttCas12a	#4	288	22	7.64	4	1.39	a
	#18	167	7	4.19	1	0.60	
	#19	295	37	12.54	7	2.37	
UdttCas12a	#1	192	16	8.33	3	1.56	a
	#4	192	15	7.81	2	1.04	
	#8	192	13	6.77	3	1.56	

GT efficiency was calculated based on the number of individual T1 transformants examined. One-way ANOVA and Tukey test were applied to analyze standard differences (SDs, *P* < 0.05) for the precise GT efficiencies

**Fig. 4:**
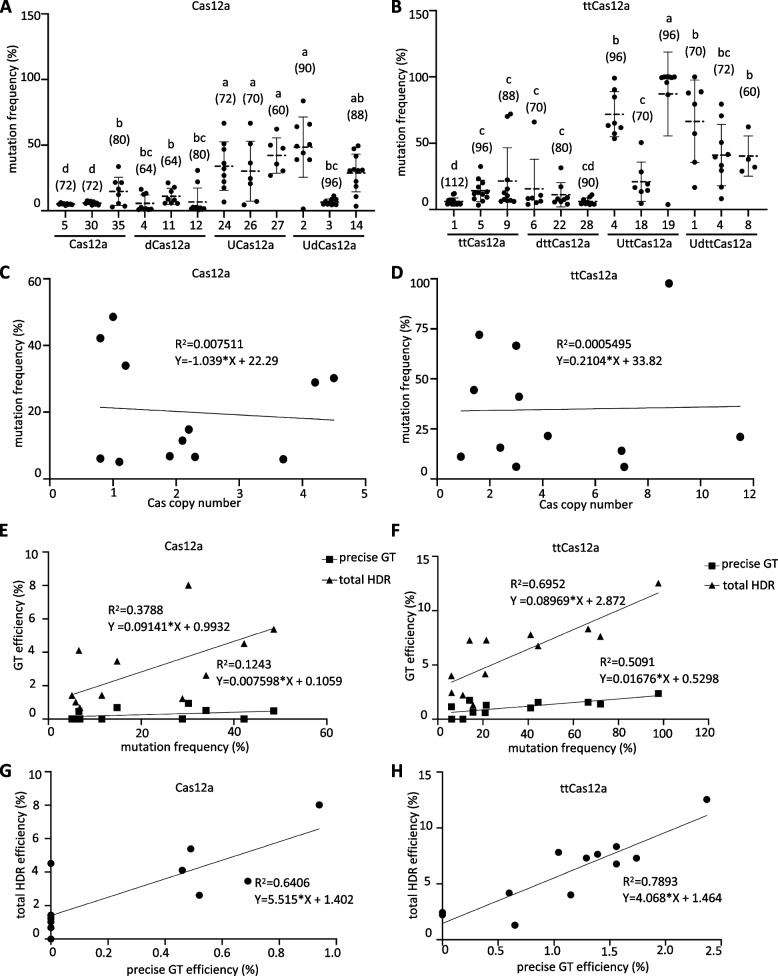
Statistical analysis of DSB and GT efficiency at the *EMB2410* locus. **A**, **B**, Mutation frequency of GT-negative *GFP*-KI at *EMB2410* locus transgenic plants in Cas12a (**A**) and ttCas12a (**B**) parental lines. Mutation frequencies were determined by PCR with full-length primers, sequencing and TIDE. The numbers in parenthesis represent the number of individual samples analyzed. The lines show mean with SD of individual values. One-way ANOVA and Tukey test were applied to analyze standard differences (*P* < 0.05). **C**, **D**, Correlation analysis of mutation frequency at *EMB2410* locus and Cas12a/ttCas12a copy number in Cas12a (**C**) and ttCas12a (**D**) parental lines. The Cas12a/ttCas12a copy number was determined by q-PCR in T1 parental line plants and calculated by the 2^−∆∆CT^ method. *Actin7* was used as an internal reference. **E**, **F**, Correlation analysis of mutation frequency and *GFP*-KI GT efficiency at the *EMB2410* locus in Cas12a (**E**) and ttCas12a (**F**) parental lines. Squares indicate precise GT, triangles indicate total HDR. **G**, **H**, Correlation analysis of precise and total HDR efficiency in Cas12a (**G**) and ttCas12a (**H**) parental lines

More detailed analyses were conducted to further evaluate the relationship between the use of enhancers and GT efficiency. First, the mutation frequency of the *EMB2410* target site in 60–112 independent GT-negative T1 transgenic plants was determined. The results demonstrated that the utilization of the AtUbq10 transcriptional enhancer markedly augmented the mutation frequency in both Cas12a and ttCas12a (Fig. 4A, B). Next, the relationship between the copy number of Cas12a and ttCas12a and the mutation frequency of the *EMB2410* target site was examined, but no correlation was found between the two (Fig. 4C, D). The screening detected a number of imprecise as well as precise GT events, as has been reported in the previous studies [25, 37]. These imprecise GT events were correctly incorporated by homology-directed repair (HDR) in one arm, but T-DNA was incorporated by non-homologous end-joining (NHEJ) in the other arm (Fig. 3B, dCas12a). In these imprecise GT events, at least one arm was correctly integrated by HDR, and these events were stably inherited. Therefore, the imprecise GT events were considered as total HDR events and statistical calculations were performed [25]. Statistical analysis of GT efficiency and mutation frequency at the *EMB2410* locus revealed a weak positive correlation for Cas12a and a strong positive correlation for ttCas12a for both total HDR and precise GT efficiency (Fig. 4E, F). Furthermore, for both Cas12a and ttCas12a, there is a significant positive correlation between the precise GT and total HDR efficiencies (Fig. 4G, H). These results indicate that DSB frequency by the CRIPR/Cas12a system could be one of the crucial factors determining GT performance.

### Base substitutions at the *ALS* locus

**Fig. 5:**
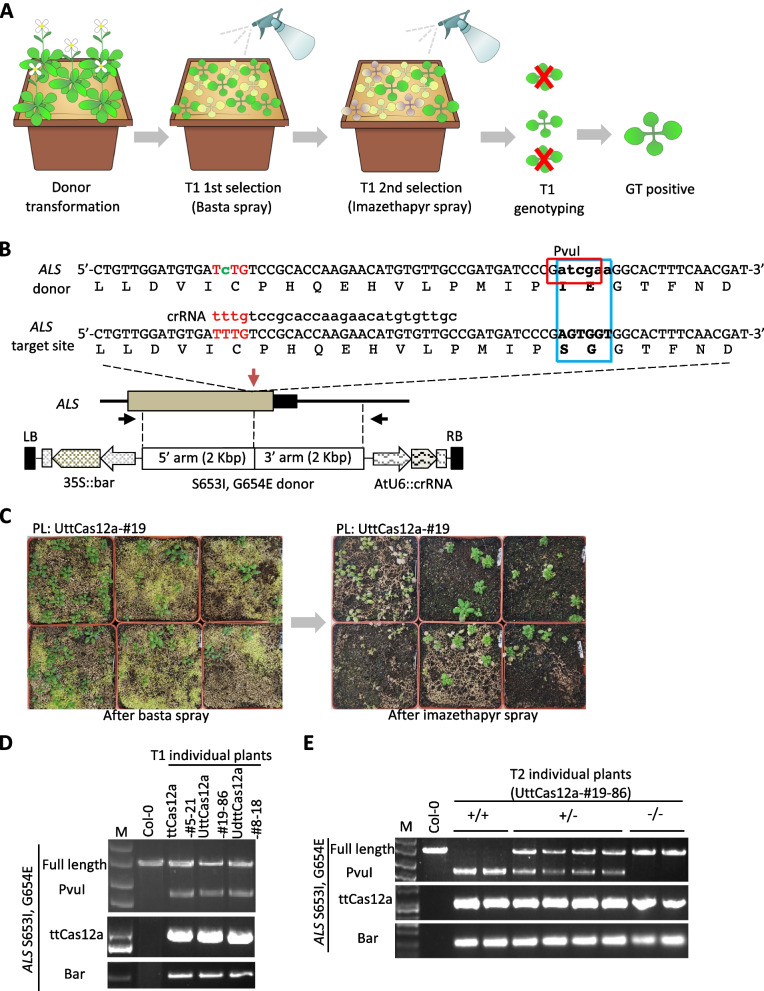
Characterization of amino acid substitution at the *ALS* locus. **A** Overview of efficient screening for amino acid substitution GT events at the *ALS* locus. T1 transformants were screened with Basta spray, followed by imazethapyr spray to screen for amino acid substitution GT events and further genotyping. **B** Detail of *ALS* substitution. The donor template harbors S653I and G654E substitutions, which also overlap the PvuI restriction enzyme site and are flanked by 2 Kbp homology arms. The black arrows represent the full-length primer for *ALS* genotyping. Red arrow indicates the target site of crRNA for the *ALS* locus. Bold font in blue box represents bases replacement and amino acid substitution. Red letters indicate PAM. **C** Phenotypes of Basta and imazethapyr spray screening. The image on the left shows the primary screening with Basta spray on T1 generation transformants. On the right is the second screening by imazethapyr spraying. **D** PCR genotyping of precise *ALS* S653I, G654E in T1 transformants. PCR-based genotyping was performed on the imazethapyr-resistant plants. Full-length primers were used for PCR followed by PvuI enzyme digestion. Three parental lines, ttCas12a-#5, UttCas12a-#19, and UdttCas12a-#8, were used for *ALS* base substitution GT by sequential transformation strategy. **E** Inheritance of the precise S653I and G654E substitution GT events at the *ALS* locus. Col-0 was used as a control. M; DNA size marker

Next, we examined whether the sequential transformation strategy with the Cas12a systems could introduce precise base substitutions as well as *GFP*-KI. The *Acetolactate synthase* (*ALS*) locus is one of the most frequently analyzed target sites for genome editing studies because of the herbicides resistance phenotype of base substitution mutants [43]. Imidazolinone (IM) herbicides inhibit ALS, an enzyme important in the biosynthesis of branched-chain amino acids in plants [44]. The donor sequence with 2 Kbp homology arms for GT was designed to introduce two amino acid substitutions (S653I and G654E) and confer imazethapyr herbicide resistance. Therefore, for efficient screening, transformed plants were subjected to Basta spraying, followed by imazethapyr herbicide application to select for GT events, which were determined by PCR (Fig. 5A). The donor sequence contained a PvuI restriction enzyme site at the S653I and G654E substitution site to distinguish the GT allele, and a T-to-C silent mutation was incorporated into the protospacer adjacent motif (PAM) sequence to avoid re-cleavage by ttCas12a (Fig. 5B). For amino acid substitution GT, three parental lines, ttCas12a-#9, UttCas12a-#19, and UdttCas12a-#8, were selected according to the results of the *EMB2410*-*GFP* KI experiment and the donor construct was transformed via *Agrobacterium tumefaciens* (Agrobacterium). After imazethapyr herbicide treatment, a large portion of the Basta-resistant transformants died (Fig. 5C). In all three parental backgrounds, at least one precise *ALS* bases substitution GT lines were obtained (Fig. 5D). All GT events were confirmed for their accuracy by Sanger sequencing. The results revealed that all intended base substitutions, including silent mutations in the PAM sequence, were correctly incorporated (Supplementary Figure S5B). The efficiency of GT ranged from 0.79–1.02% based on the number of individual Basta-resistant T1 transformants (Table 2). GT ratios were comparable among the different parental lines. This may be due to plateaued DSB and GT frequencies in selected parental lines that exhibited the highest GT efficiency at the *EMB2410* locus. All precise amino acid substitution GT events were inherited by the progenies according to Mendelian inheritance (Fig. 5E). Overall, these results indicate that the Cas12a system can be applied not only to KI but also to amino acid substitution GT in plants.

**Table 2:** Amino acid substitution GT efficiencies at the *ALS* locus

Parental line	Basta spray	Imazethapyr spray	Precise GT	Precise GT efficiency to basta spray (%)	Precise GT efficiency to Imazethapyr spray (%)
ttCas12a—#9	202	22	2	0.99	9.09
UttCas12a—#19	98	13	1	1.02	7.69
UdttCas12a—#8	127	19	1	0.79	5.26

GT efficiency was calculated based on the number of individual T1 transformants examined

## Discussion

In this study, we performed a detailed characterization of the sequential transformation approach previously developed by our group to generate GT in plants via HDR, using the CRISPR/Cas12a systems instead of Cas9 [25, 31, 36, 37]. While GT in plants using all-in-one strategies via Cas12a and ttCas12a have been reported [11, 13, 42, 45], this study showed that efficient GT events, including precise KIs and base substitutions by sequential transformation strategy, are possible at two loci in Arabidopsis. Moreover, this study demonstrates that the employment of enhancers alone and in combinations for the Cas12a systems can increase mutagenesis efficiency, although not always resulting in GT. Nevertheless, despite the implementation of these modifications, the anticipated enhancement in GT efficiency could not be achieved in the present study.

Precise and heritable GT is a useful tool for molecular research and molecular breeding. However, the establishment of GT remains a difficult task, especially in seed plants, due to the extremely low efficiency of homologous recombination and the difficulty of delivering donor templates [6, 10, 46]. Various approaches have been tried to improve GT efficiency via engineered SSNs in plants. The most simple and effective way to improve the efficiency of GT via HDR is to promote the frequency of DSB. For example, the use of a highly efficient CRISPR/Cas system, such as ttCas12a, which has exhibited higher mutational and GT efficiencies than unmodified Cas12a [12, 13]. Our sequential transformation strategy is also a method to promote DSB activity. This is because higher levels of Cas9 activity can be maintained by using highly efficient parental lines [36, 37]. In previous reports, GT by sequential transformation strategy has used exclusively one parental line, CS69955, generated by our colleagues, which harbors DD45 pro::Cas9 with an sgRNA targeting the *GL2* locus [25, 37, 38]. This means that the Cas9 parental lines have not been evaluated and optimized for efficient GT for sequential transformation strategy. Therefore, to efficiently establish GT, a series of Cas12a parental lines with and without enhancers were generated and characterized in this study. The results showed that ttCas12a exhibited higher DSB frequency than Cas12a and that the transcriptional enhancer AtUbq10 5' UTR with the first intron promotes these DSB efficiencies. In contrast to our initial expectations, the utilization of the translational enhancer dMac3 did not result in an improvement in DSB and GT frequencies, and in fact, exhibited a frequency that was lower than that observed in the absence of the enhancer. One possibility is that the combination with the Cas12a systems is not suitable for the dMac3 enhancer. The most important and interesting finding is that the efficiency of DSB and GT in the second transformation is improved in the parental lines with the enhancers, even though all selected parental lines showed similar mutation frequencies in these T1 generations. This indicates that the potential for DSB activity, already saturated and unable to detect such differences in the T1 generation of parental lines, was magnified by the use of enhancers.

It has been reported that the ttCas12a variant containing ten introns showed higher GT efficiency than the version without introns [22]. Intron splicing is considered to facilitate the transport of mature mRNA into the cytoplasm, promote mRNA stability, and consequently increase the amount of translation products [15, 47–49]. The AtUbq10 first intron in this study may function in the same way as the introns in the previous report, promoting Cas12a and ttCas12a mRNA stability and increasing translation efficiency. The results clearly indicate that the employment of enhancers increases the DSB frequency and consequently the GT efficiency. In addition, this study showed a strong, statistically significant positive correlation between the DSB ratio and GT efficiency. These results together indicate that DSB by engineered SSNs such as Cas12a is one of the most critical factors determining GT efficiency via HDR. In this study, the enhancers demonstrated an improvement in the efficiency of GT, although the effect was not as pronounced as anticipated. One potential explanation for this discrepancy is that the DSB required for establishing GT may have reached a plateau. Previous research has indicated that a higher DSB efficiency does not necessarily correspond to a higher GT frequency [50, 51]. These findings indicate that additional factors, in addition to DSB frequency, are necessary for efficient GT establishment. These factors include cell cycle timing and those required for HDR. Further studies are warranted to elucidate the precise role of these factors in GT establishment.

Although previous reports and the present study indicate that the DSB frequency of engineered SSNs is one of the most important determinants of GT efficiency, the molecular mechanisms underlying GT via HDR remain largely unknown [6, 22, 25, 37]. In this study, not only precise GT events were detected, but also imprecise GT events. The imprecise GT events are the integration of T-DNA fragments by NHEJ in one arm and by HDR in the other arm, which is consistent with previous reports [25, 37, 42, 50, 52–54]. Based on these imprecise GT events, a hypothetical working model has been proposed in which single-stranded T-DNA (also known as T-strand) released from Agrobacterium is the most likely repair donor template for HDR-mediated GT in plants [6, 37]. Indeed, a high frequency of T-DNA integration into CRISPR/Cas9 cleavage sites has been reported [55], indicating the presence of the single-stranded T-DNA near the DSB site, which could be used as a donor template for HDR. In addition, a Cas9-VirD2 fusion protein that can bind to single-stranded T-DNA has been reported to improve GT efficiency in rice [56]. These findings would further support our hypothetical working model.

Imprecise GT events, in which one arm is incorporated by HDR and the other by NHEJ, were observed in this and previous studies to be more frequent than precise GT [25, 32, 37]. The reported NHEJ-mediated entire integration of T-DNA into the DSB sites [55] could not be detected in our study because of the primer design, but would be likely to occur in our case as well. These results also indicate that NHEJ is the predominant repair mechanism, with or without T-DNA incorporation, and that HDR is rare even though a repair donor template is provided. Therefore, how to improve HDR efficiency, not DSB frequency, will be the main subject for GT improvement in the future.

## Conclusions

In this study, efficient GT via the Cas12a system in Arabidopsis was demonstrated by using sequential transformation strategy, but no comparison was made with the all-in-one strategy. Our previous reports have shown that the application of a sequential transformation strategy is more efficient for GT than an all-in-one strategy [36, 37]. Therefore, this study aimed to further improve the efficiency of GT by applying enhancers and establishing optimized parental lines in a sequential transformation strategy via the Cas12a system. The results revealed that the combination of ttCas12a and AtUbq10 first intron transcriptional enhancer lines are the best parental lines for GT establishment by a sequential transformation strategy. Taken together, these results suggest that the improvement of GT by increasing DSB frequency has reached a plateau and no further improvement can be expected. In the present study, we demonstrated that Cas12a-mediated GT is comparable in efficiency to Cas9-mediated GT in previous reports [25, 37]. Therefore, targeting a wider range of genomic sequences, such as sequences for which the appropriate Cas9 sgRNA cannot be designed, allows the desired genome editing to be performed accurately. Accordingly, the results of this study can be widely applied to basic molecular research and molecular breeding not only in Arabidopsis but also in other plant species.

## Materials and methods

### Gene accession numbers

*EMB2410*, At2g25660; *ALS*, At3g48560; *AtUbq10*, At4g05320; *Actin7*, At5g09810.

### Plant materials and growth condition

*Arabidopsis thaliana* (Col-0) for parental line generation was germinated on 1/2 Murashige & Skoog (MS) solid medium and cultured in incubator for two weeks, then transplanted seedlings into soil in the greenhouse. The growth condition is light/16 h and dark/8 h at 22˚C. The generated parental lines were germinated on 1/2 MS medium with 50 mg/L hygromycin. A DD45 pro::Cas9 parental line (ABRC stock CS69955) was used as a control for hygromycin resistance. The T1 transformants after sequential transformation were directly germinated in the soil. One week later, 0.2% Basta solution was used for Basta spray. For *ALS* substitution, 2 mg/L imazethapyr was used for screening after 3–4 times Basta spray.

### Plasmid construction

All destination vectors for parental lines and sequential transformations were constructed according to the publications [36, 57]. In this study, we used the previously reported human codon-optimized *Lachnospiraceae bacterium ND2006* Cas12a (previously known as LbCpf1) [58]. The ttCas12a used in this study was prepared by introducing the D156R amino acid substitution into a human codon-optimized Cas12a according to the previous report [12]. The DNA fragments of each construct were amplified by high fidelity DNA polymerase Phanta Max (Vazyme, Nanjing, China) and purified the gel using FastPure Gel DNA Extraction Mini Kit (Vazyme, Nanjing, China). crRNAs were designed by using the online site CRISPOR (http://crispor.tefor.net/) [59] (Supplementary Table S1). The AtU6-26 promoter was used to drive crRNA expression. For the parental line, DD45 promoter driven Cas12a or ttCas12a with or without enhancers constructs were generated in the pCambia1300 background. For donor construct, the donor sequence was cloned into pCambia3301 background for sequential transformation. Ligation products were transformed into *E. coli*, monoclonal bacteria were detected by PCR, recombinant plasmids were extracted and sequenced. All primers used in this study are listed in Supplementary Table S2.

### Transformation of Agrobacterium and Arabidopsis plant

The prepared destination vectors were transferred to Agrobacterium (*Agrobacterium tumefaciens*) GV3101 competent cells by heat shock method. Briefly, the constructed plasmid (approximately 100 ng) was added to 50–100 µl Agrobacterium GV3101 competent cells and incubated for 25 min on ice, 5 min in liquid nitrogen, 5 min heat shock at 37˚C, and 5 min on ice. Then, added 600 µl liquid LB and incubated at 28˚C, 200 rpm for 2–3 h. The liquid culture was centrifuged at 4000 rpm, 500 µl of the LB supernatant was discarded, and the pellet was resuspended. Spread Agrobacterium on the LB plates with 50 mg/L kanamycin and rifampicin for 2–3 days at 28˚C.

Freshly transformed Agrobacterium was used for plant transformation. After pre-culturing the transformed Agrobacterium in 5 mL liquid LB, cultured in 100 mL liquid LB medium containing 50 mg/L kanamycin and rifampicin until the OD_600_ reaches about 0.6–0.8, and collected by centrifugation at 4000 rpm, 4 ˚C for 20 min. The collected Agrobacterium was resuspended in 100 mL infection solution containing 5% (w/v) sucrose, 0.22% (w/v) MS and 0.05% (v/v) Silwet-77. The flowers of Arabidopsis were soaked in the transformation buffer for 30 s and cultured in dark at 22˚C for 16–24 h, then cultivated the plants continued with light/16 h and dark/8 h at 22˚C.

### Genomic DNA extraction

The total genomic DNA was extracted from leaf tissue by the cethyltrimethyl ammonium bromide (CTAB) method for individual plant analysis. Leaf tissues were ground into fine powder in liquid nitrogen using ShakeMaster AUTO (Bio Medical Science Inc., Tokyo, Japan). Extracted DNA was used for the following DNA analyses.

### T-DNA Copy number

The Cas12a and ttCas12a copy number was determined by qPCR using gDNA as template. SYBR Green Super Mix (Bio-Rad Laboratories, Hercules, USA) was used for the qPCR reaction system. *Actin7* gene was used as an internal reference gene. The Cas12a and ttCas12a copy number was calculated by the 2^−∆∆CT^ method.

### Mutation frequency analysis

The T7 endonuclease I (T7EI) digestion assay was used to determine mutation frequency of all parental lines. Amplify a fragment of approximately 500 bp at the target site, denature the reaction solution at 95˚C for 5 min, renature the reaction system at room temperature, add 0.2 µl T7EI, and incubate at 37˚C for 30 min. T7EI-treated digestion products were analyzed by 2% (w/v) agarose gel electrophoresis, and relative mutation frequencies were calculated from band intensities using Image Lab (Bio-Rad Laboratories, Hercules, USA).

Alternatively, the mutation frequency of the *EMB2410* target locus was determined by PCR followed by sequencing and TIDE analysis (https://tide.nki.nl) [60]. Total DNA was extracted from a pool of 10–20 independent GT-negative T1 transgenic plants, and PCR was performed using extracted DNA as template.

### Detection of GT events

PCR analysis using specific and full-length primer sets were used to genotype the *EMB*-*GFP* KI, followed by sequence confirmation. Genotyping PCR was performed using 2 × Taq Plus Master Mix II (Vazyme, Nanjing, China) according to the manufacturer's instructions. Genotyping PCR products were electrophoresed on 1.5% (w/v) agarose gels and visualized with Image Lab software (Bio-Rad Laboratories, Hercules, USA). For genotyping of *ALS* substitutions, full-length primers were used for PCR and PCR products were identified by PvuI restriction enzyme digestion. And the base substitution GT events at the *ALS* locus were confirmed by Sanger sequencing.

### Supplementary Information

Additional file 1: Supplementary Table S1. Prediction scores for crRNAs. The crRNAs activities were predicted by using CRISPOR online website (http://crispor.tefor.net/). Out-of-Frame score, only for deletions. Predicts the percentage of clones that will carry out-of-frame deletions, based on the micro-homology in the sequence flanking the target site. Supplementary Table S2. Sequences of primers. Supplementary Figure S1. crRNA design in the intergenic region for parental lines. The crRNA was designed at the intergenic region where the two genes, At1g53990 and At1g54000, are located in the tail-to-tail direction. The black arrows indicate the primers used for T7EI digestion assay. The red underline marks crRNA, and bold red represents PAM. Supplementary Figure S2. Evaluation of Cas12a candidate parental lines in T1. A, B, C, D, Bar chart of mutation frequency at the target intergenic region and copy number of Cas12a (A), dCas12a (B), UCas12a (C) and UdCas12a (D) T1 individual candidate parental lines. The histogram is plotted in order of mutation frequency. The white columns represent the mutation frequency at the target intergenic region, the gray columns represent the Cas12a copy number. Asterisks and yellow columns indicate plant lines used as parental lines for sequential transformation. The mutation frequencies of the target intergenic region in the parental lines are indicated. E, F, G, H, Correlation analysis of mutation frequency at the target intergenic region and copy number of Cas12a (E), dCas12a (F), UCas12a (G) and UdCas12a (H). The filled circles represent independent parental lines. The T7EI digestion assay was used to detect the mutation frequencies of intergenic targeting site. The Cas12a copy number was determined by q-PCR in each T1 parental line plants and calculated by the 2^−∆∆CT^ method. *Actin7* was used as an internal reference. Supplementary Figure S3. Evaluation of ttCas12a candidate parental lines in T1. A, B, C, D, Bar chart of mutation frequency at the target intergenic region and copy number of ttCas12a (A), dttCas12a (B), UttCas12a (C) and UdttCas12a (D) T1 individual parental line. The histogram is plotted in order of mutation frequency. The white columns represent the mutation frequency at the target intergenic region, the gray columns represent the ttCas12a copy number. Asterisks and yellow columns indicate plant lines used as parental lines for sequential transformation. The mutation frequencies of the target intergenic region in the parental lines are indicated. E, F, G, H, Correlation analysis of mutation frequency at the target intergenic region and ttCas12a copy number in ttCas12a (E), dttCas12a (F), UttCas12a (G) and UdttCas12a (H). The filled circles represent different parental lines. The T7EI digestion assay was used to detect the mutation frequencies of intergenic targeting site. The ttCas12a copy number was determined by q-PCR in each T1 parental line plants and calculated by the 2^−∆∆CT^ method. *Actin7* was used as an internal reference. Supplementary Figure S4. Hygromycin-resistant phenotypes in the Cas12a/ttCas12a T2 individual candidate parental lines. A, B, Hygromycin resistance of Cas12a (A) and ttCas12a (B) T2 candidate parental lines. Labeled plant lines are the parental lines selected for sequential transformation. Supplementary Figure S5. Confirmation of precise GT events by sequencing. A, B, Sequence confirmation of *GFP*-KI at the *EMB2410* locus (A) and amino acid substitutions at the *ALS* locus (B). PCR of the *EMB2410* and *ALS* target loci was performed using full-length primers. The amplified PCR products were sequenced to confirm the precise *GFP*-KI and *ALS* S653I, G654E substitutions at the *EMB2410* and *ALS* target loci, respectively. Lowercase letters indicate the 3' UTR of *EMB2410*, black squares indicate the terminator, and bold letters indicate the crRNA sequence. Supplementary Figure S6. Original uncropped gel pictures.

## Data Availability

The authors declare that all the data supporting the findings of this study are available within the manuscript. The data sets generated or analyzed during the current study are available from the corresponding author on reasonable request.
