# Supplementary material for: Cas12a-mediated gene targeting by sequential transformation strategy in Arabidopsis thaliana
Source: BMC Plant Biol. 2024 Jul 12;24:665. doi: 10.1186/s12870-024-05375-z (PMC11241819; doi:10.1186/s12870-024-05375-z)
Supplement: Supplementary file 1 — Additional file 1: Supplementary Table S1. Prediction scores for crRNAs. The crRNAs activities were predicted by using CRISPOR online website (http://crispor.tefor.net/). Out-of-Frame score, only for deletions. Predicts the percentage of clones that will carry out-of-frame deletions, based on the micro-homology in the sequence flanking the target site. Supplementary Table S2. Sequences of primers. Supplementary Figure S1. crRNA design in the intergenic region for parental lines. The crRNA was designed at the intergenic region where the two genes, At1g53990 and At1g54000, are located in the tail-to-tail direction. The black arrows indicate the primers used for T7EI digestion assay. The red underline marks crRNA, and bold red represents PAM. Supplementary Figure S2. Evaluation of Cas12a candidate parental lines in T1. A, B, C, D, Bar chart of mutation frequency at the target intergenic region and copy number of Cas12a (A), dCas12a (B), UCas12a (C) and UdCas12a (D) T1 individual candidate parental lines. The histogram is plotted in order of mutation frequency. The white columns represent the mutation frequency at the target intergenic region, the gray columns represent the Cas12a copy number. Asterisks and yellow columns indicate plant lines used as parental lines for sequential transformation. The mutation frequencies of the target intergenic region in the parental lines are indicated. E, F, G, H, Correlation analysis of mutation frequency at the target intergenic region and copy number of Cas12a (E), dCas12a (F), UCas12a (G) and UdCas12a (H). The filled circles represent independent parental lines. The T7EI digestion assay was used to detect the mutation frequencies of intergenic targeting site. The Cas12a copy number was determined by q-PCR in each T1 parental line plants and calculated by the 2−∆∆CT method. Actin7 was used as an internal reference. Supplementary Figure S3. Evaluation of ttCas12a candidate parental lines in T1. A, B, C, D, Bar chart of mutation freq [file 12870_2024_5375_MOESM1_ESM.pdf]

Supplementary Figure Legends

Supplementary Figure S1. crRNA design in the intergenic region for parental lines.

The crRNA was designed at the intergenic region where the two genes, At1g53990 and At1g54000, are located in the tail-to-tail direction. The black arrows indicate the primers used for T7EI digestion assay. The red underline marks crRNA, and bold red represents PAM.

Supplementary Figure S2. Evaluation of Cas12a candidate parental lines in T1.

A, B, C, D, Bar chart of mutation frequency at the target intergenic region and copy number of Cas12a (**A**), dCas12a (**B**), UCas12a (**C**) and UdCas12a (**D**) T1 individual candidate parental lines. The histogram is plotted in order of mutation frequency. The white columns represent the mutation frequency at the target intergenic region, the gray columns represent the Cas12a copy number. Asterisks and yellow columns indicate plant lines used as parental lines for sequential transformation. The mutation frequencies of the target intergenic region in the parental lines are indicated. **E, F, G, H,** Correlation analysis of mutation frequency at the target intergenic region and copy number of Cas12a (**E**), dCas12a (**F**), UCas12a (**G**) and UdCas12a (**H**). The filled circles represent independent parental lines. The T7EI digestion assay was used to detect the mutation frequencies of intergenic targeting site. The Cas12a copy number was determined by q-PCR in each T1 parental line plants and calculated by the $2^{-\Delta\Delta CT}$ method. *Actin7* was used as an internal reference.

Supplementary Figure S3. Evaluation of ttCas12a candidate parental lines in T1.

A, B, C, D, Bar chart of mutation frequency at the target intergenic region and copy number of ttCas12a (**A**), dttCas12a (**B**), UttCas12a (**C**) and UdttCas12a (**D**) T1 individual parental line. The histogram is plotted in order of mutation frequency. The white columns represent the mutation frequency at the target intergenic region, the gray columns represent the ttCas12a copy number. Asterisks and yellow columns indicate plant lines used as parental lines for sequential transformation. The mutation frequencies of the target intergenic region in the parental lines are indicated. **E, F, G, H,** Correlation analysis of mutation frequency at the target intergenic region and ttCas12a copy number in ttCas12a (**E**), dttCas12a (**F**), UttCas12a (**G**) and UdttCas12a (**H**). The

filled circles represent different parental lines. The T7EI digestion assay was used to detect the mutation frequencies of intergenic targeting site. The ttCas12a copy number was determined by q-PCR in each T1 parental line plants and calculated by the $2^{-\Delta\Delta CT}$ method. *Actin7* was used as an internal reference.

Supplementary Figure S4. Hygromycin-resistant phenotypes in the Cas12a/ttCas12a T2 individual candidate parental lines.

A, B, Hygromycin resistance of Cas12a (A) and ttCas12a (B) T2 candidate parental lines. Labeled plant lines are the parental lines selected for sequential transformation.

Supplementary Figure S5. Confirmation of precise GT events by sequencing.

A, B, Sequence confirmation of *GFP*-KI at the *EMB2410* locus (**A**) and amino acid substitutions at the *ALS* locus (**B**). PCR of the *EMB2410* and *ALS* target loci was performed using full-length primers. The amplified PCR products were sequenced to confirm the precise *GFP*-KI and *ALS* S653I, G654E substitutions at the *EMB2410* and *ALS* target loci, respectively. Lowercase letters indicate the 3' UTR of *EMB2410*, black squares indicate the terminator, and bold letters indicate the crRNA sequence.

Supplementary Figure S6. Original uncropped gel pictures.

Supplementary Table S1. Prediction scores for crRNAs.

The crRNAs activities were predicted by using CRISPOR online website (http://crispor.tefor.net/). Out-of-Frame score, only for deletions. Predicts the percentage of clones that will carry out-of-frame deletions, based on the micro-homology in the sequence flanking the target site.

Targeting locus	crRNA	Predicted Efficiency	Out-of-Frame
Intergenic region	*TTTG* CCACAAGACGTCTCTAATATTTC	79	71
EMB2410	*TTTG* GTGACTACCGACAAGAAAAATTA	91	71
ALS	*TTTG* TCCGCACCAAGAACATGTGTTGC	62	66

Supplementary Table S2. Sequences of primers

**Primer name**	Primer sequence (5' - 3')
**Construction**	
EMB-5'arm-F	GGCATGGCTCATCCAAAATTTATCAAACC
EMB-5'arm-R	GTCTTGTGATGTAGCAGAGTATTCAAAAAGC
GFP-F	ATGGTGAGCAAGGGCGAGGAG
GFP-R	TTACTTGTACAGCTCGTCCATGCCGT
EMB-3'arm-F	GGTGACTACCGACAAGAAAAATTATTTTATTATTTG
EMB-3'arm-R	GCACGATGTAAACGGAAATGGCG
ALS-5'arm-F	CGGCGGCAACAACAACAACAAC
ALS-5'arm-R	TTGGTGCGGACAgATCACATCCA
ALS-3'arm-F	TGGATGTGATcTGTCCGCACCAA
ALS-3'arm-R	ATTTTTTTTTTATGAACGTGCTATGGAAAATAAAGGTTGATCC
**DNA analysis for parental line**	
Cas12a/ttCas12a copy-qF1	CCTTCACCGGCTTCTTTGATA
Cas12a/ttCas12a copy-qR1	CGGGTCAGATTCTCGTTGATAG
Actin7-qF	CGTTTCGCTTTCCTTAGTGTTAGCT
Actin7-qR	AGCGAACGGATCTAGAGACTCACCTTG
intergenic-T7EI-F	ATGAAATACTAAGGTACAAAGACA
intergenic-T7EI-R	TACAGTCTCACACATCAACACATA
**Genotyping for GT**	
EMB-full length-F	CCAAATATGGATATCCGTCTGAGT
EMB-full length-R	TTCGATGATTTGGGGACAAGGT
GFP-specific-F	GGTGAACTTCAAGATCCGCC
GFP-specific-R	GCAGATGAACTTCAGGGTCAG
Cas12a-check-F	TGTCTGTGGTGGAGAAGCTG
Cas12a-check-R	GGGTGTGCCGTGAGACTTAT
Bar-check-F	TGCACCATCGTCAACCACTAC
Bar-check-R	TCAGCAGGTGGGTGTAGAGC
ALS-full length-F	TTAATGTCTGAGAAAGGCGAAAG
ALS-full length-R	TTCTCAGCCACAAATTCTACATTT
**Sequencing**	
EMB-seq-F	AAAGATTCTGAGATGGCAATGCA
EMB-seq-R	AACTGAAGTATCCAGAAGAGCCA

ALS-seq-R	CGTGTAGAGAATCTTCCAGTGAA

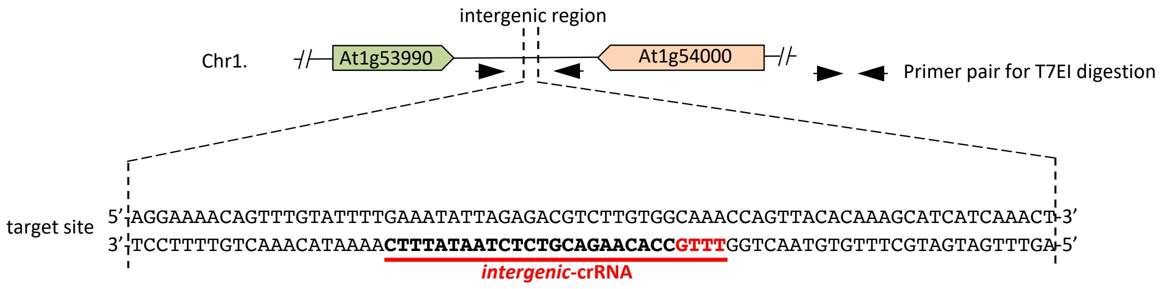

Supplementary Figure S1. crRNA design in the intergenic region for parental lines.

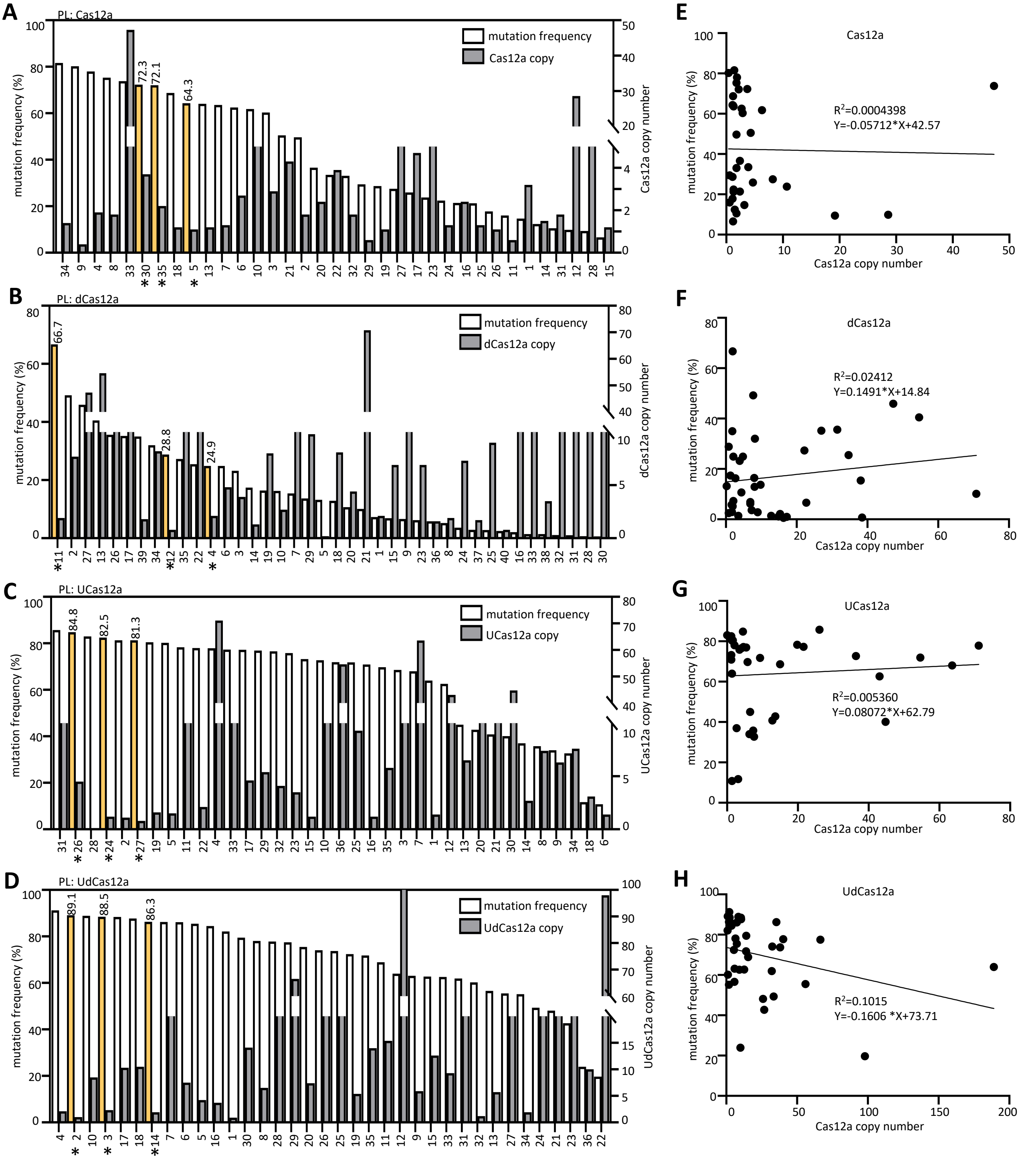

Supplementary Figure S2. Evaluation of Cas12a candidate parental lines in T1.

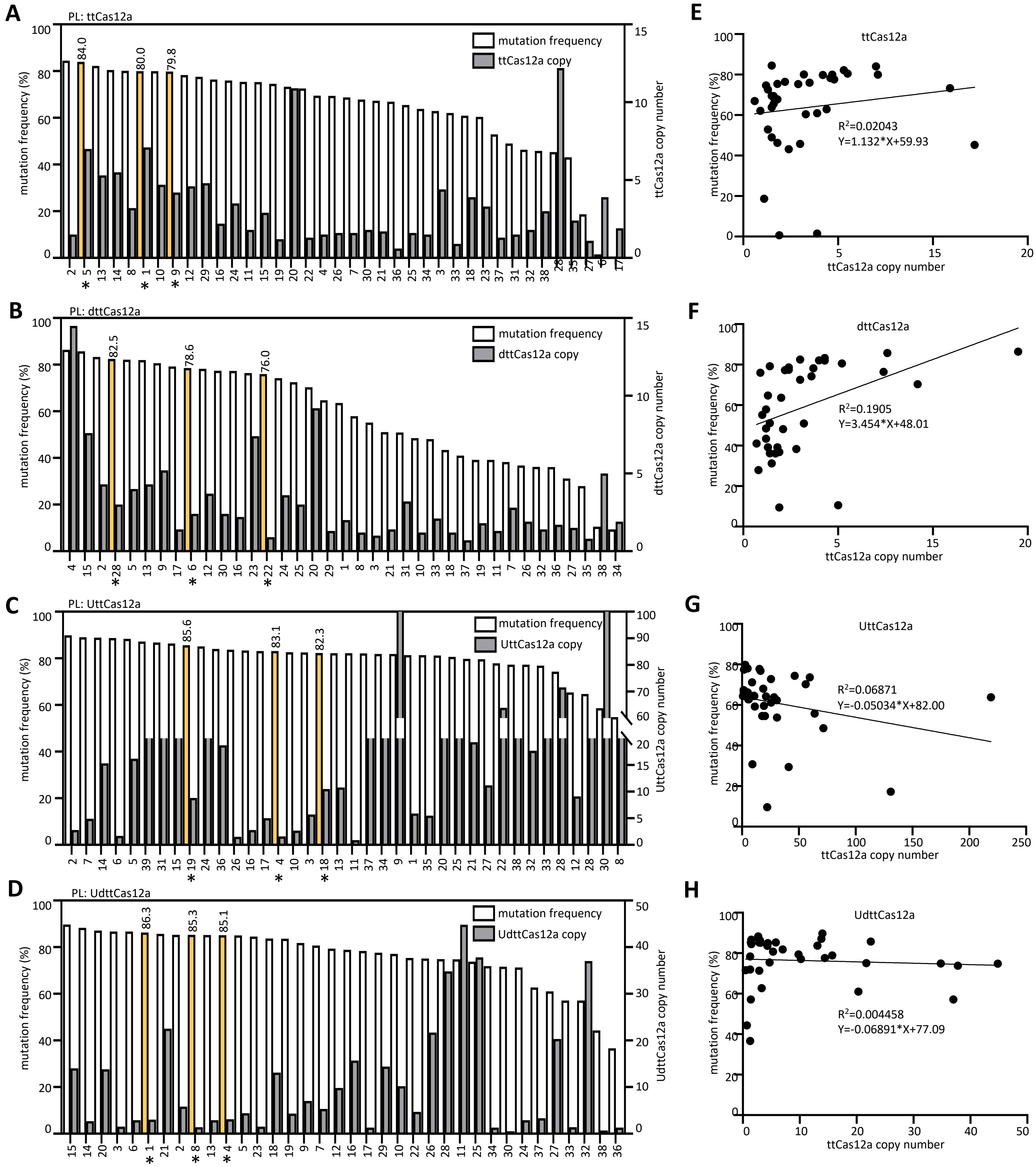

Supplementary Figure S3. Evaluation of ttCas12a candidate parental lines in T1.

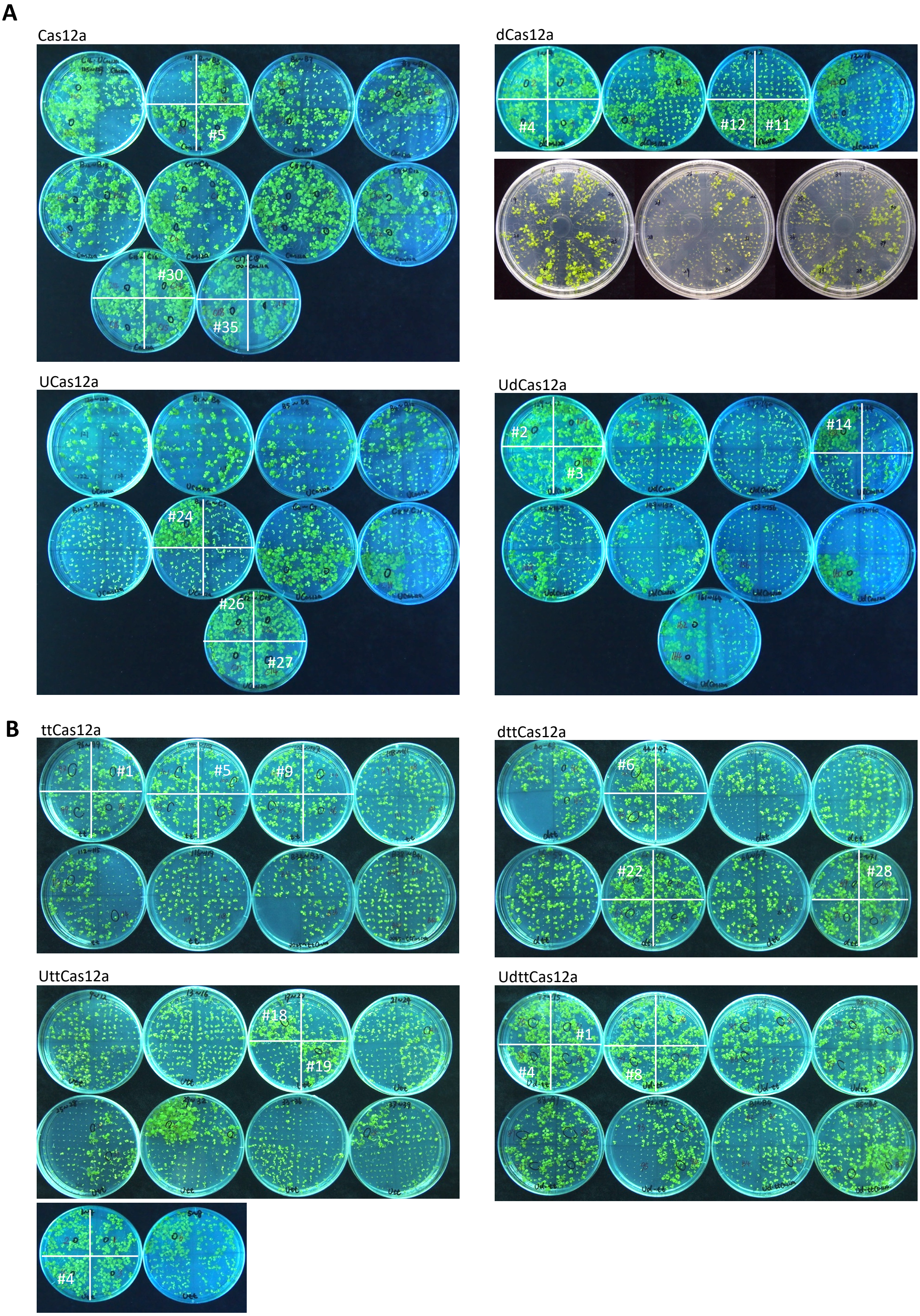

Supplementary Figure S4. Hygromycin-resistant phenotypes in the Cas12a/ttCas12a T2 individual candidate parental lines.

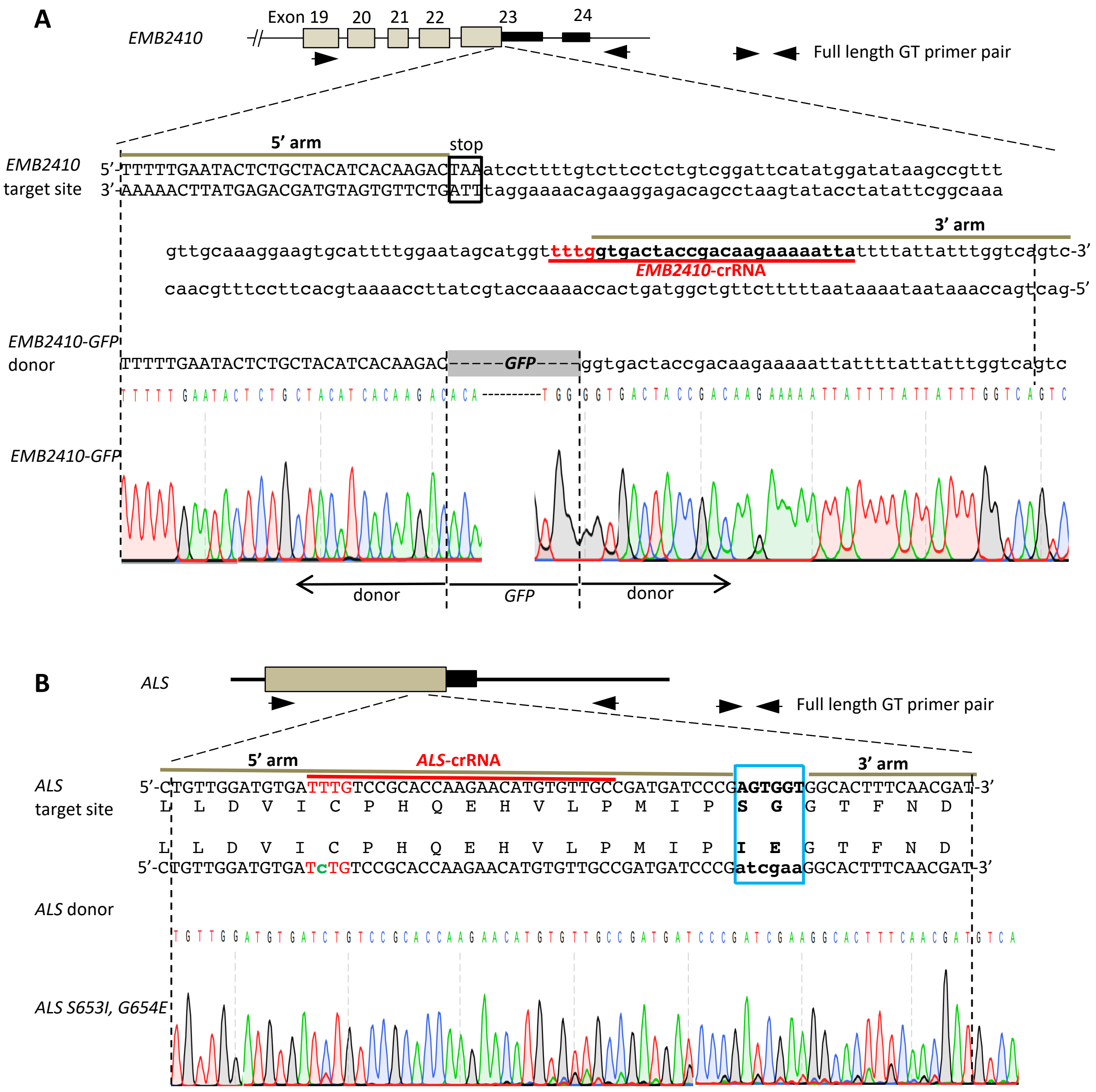

Supplementary Figure S5. Confirmation of precise GT events by sequencing.

Figure 3B

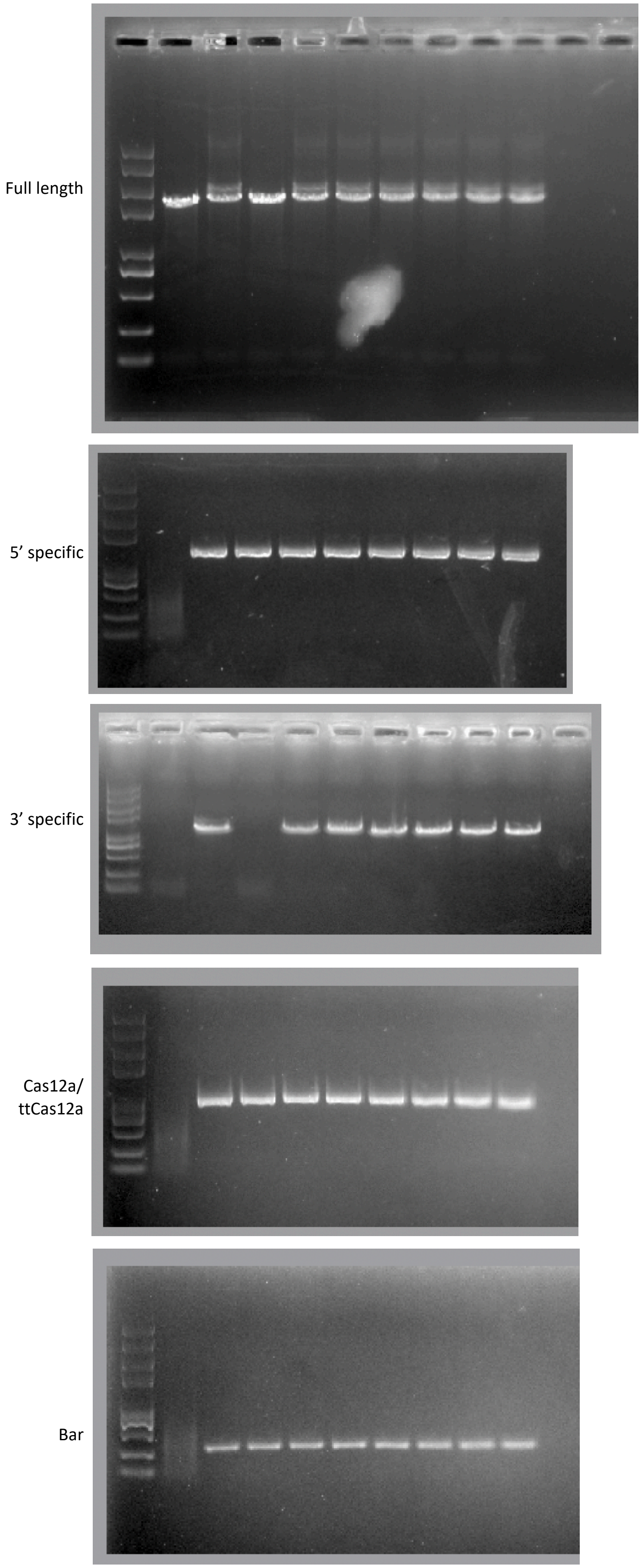

Figure 3D

Full length

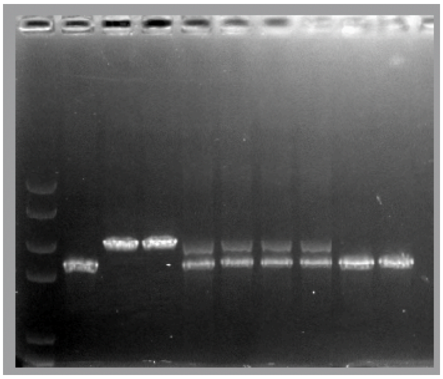

5’ specific

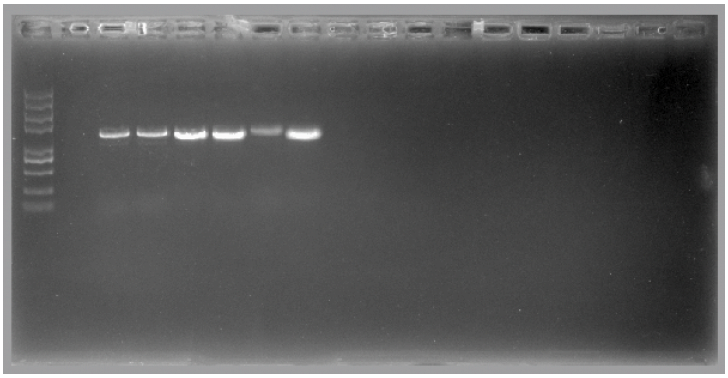

3’ specific

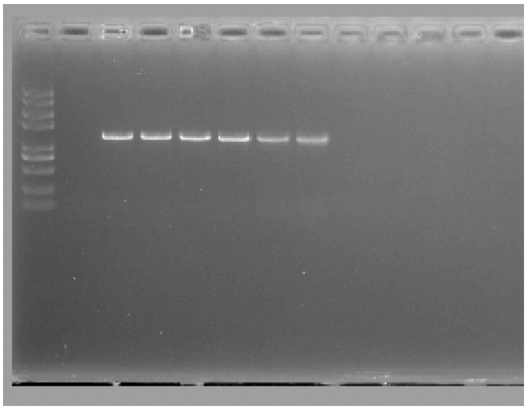

ttCas12a

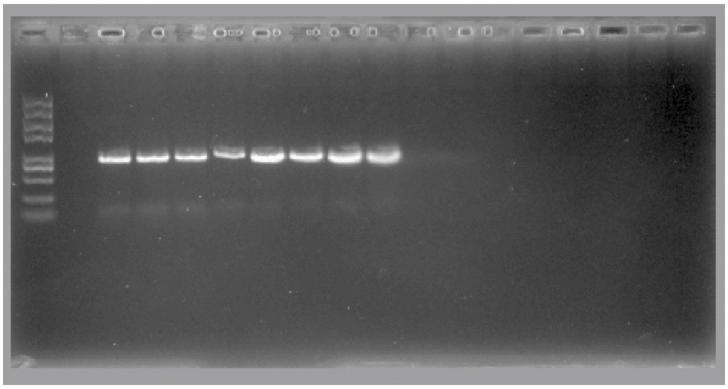

Bar

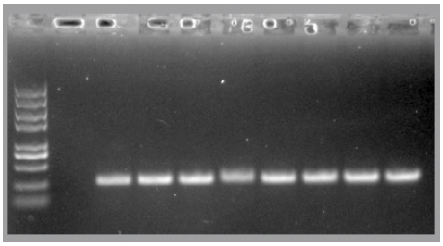

Figure 5D

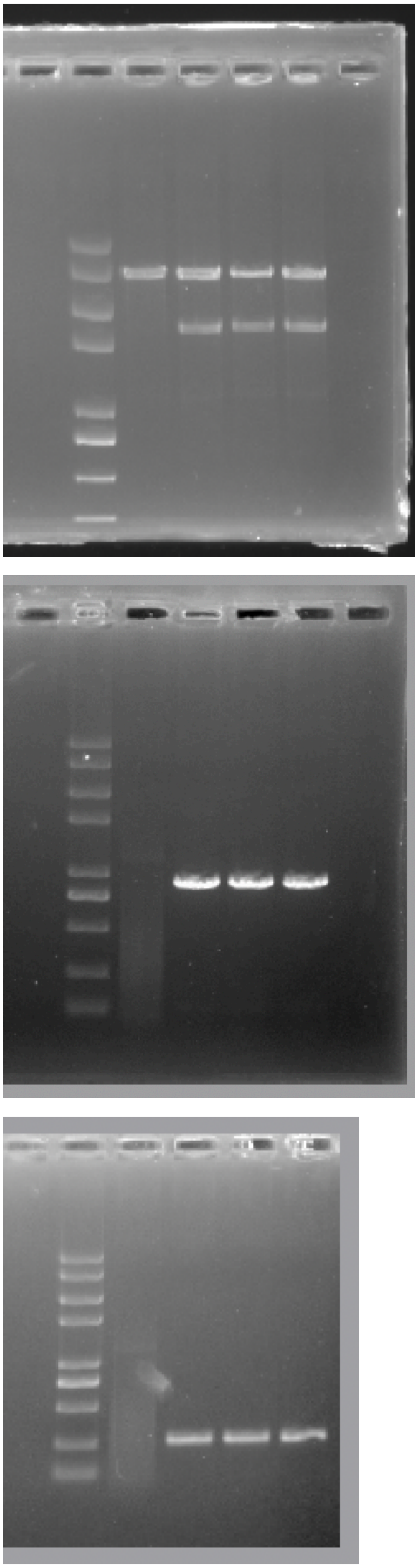

Full length

PvuI

ttCas12a

Bar

Figure 5E

Full length

PvuI

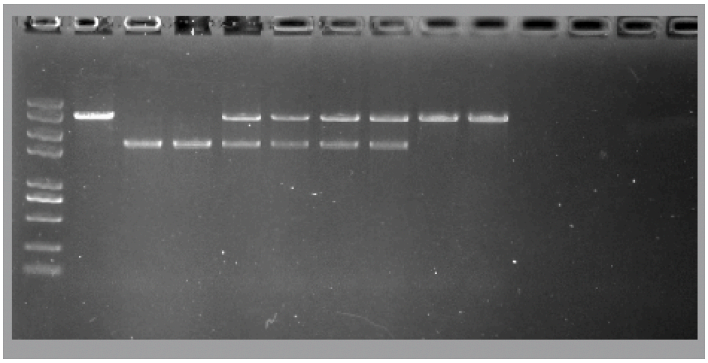

ttCas12a

Bar

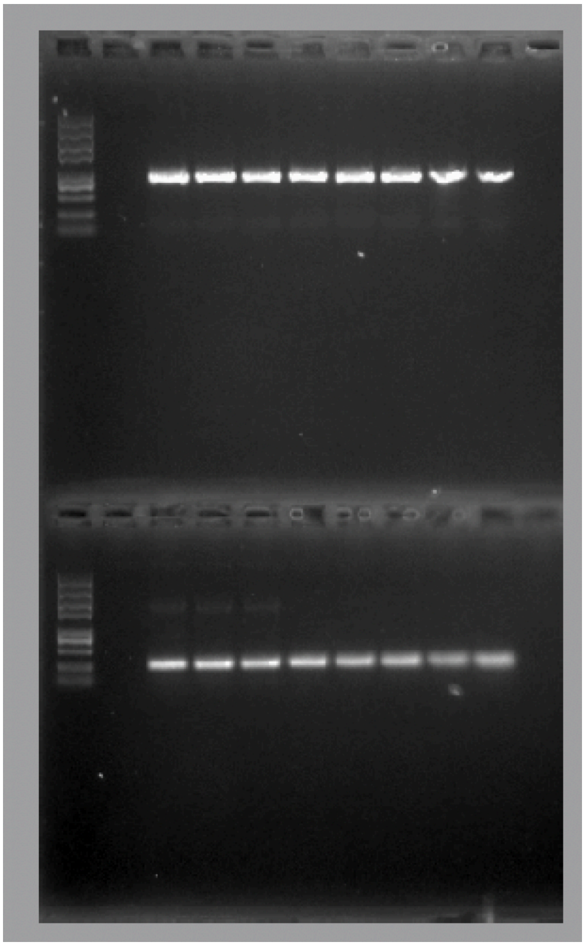

Supplementary Figure S6. Original uncropped gel pictures.
